# Growing Resilience through Interaction with Nature: Can Group Walks in Nature Buffer the Effects of Stressful Life Events on Mental Health?

**DOI:** 10.3390/ijerph16060986

**Published:** 2019-03-19

**Authors:** Melissa R. Marselle, Sara L. Warber, Katherine N. Irvine

**Affiliations:** 1 Department of Ecosystem Services, Helmholtz Centre for Environmental Research—UFZ, Permoserstr. 15, 04318 Leipzig, Germany; 2 German Centre for Integrative Biodiversity Research (iDiv) Halle-Jena-Leipzig, Deutscher Platz 5e, 04103 Leipzig, Germany; 3 Department of Family Medicine, University of Michigan, Ann Arbor, MI 48104, USA; swarber@med.umich.edu; 4 European Centre for Environment and Human Health, University of Exeter School of Medicine, Truro TR1 3HD, UK; 5 Social, Economic and Geographical Sciences, The James Hutton Institute, Craigiebuckler, Aberdeen AB15 8QH, UK; Katherine.Irvine@hutton.ac.uk

**Keywords:** stress buffering, moderation, depression, nature walks, health promotion

## Abstract

Nature-based activities have been used as therapeutic interventions for those experiencing stress and mental ill health. This study investigates whether group walks could be a nature-based intervention to foster resilience, by buffering the effects of recent stressful life events on mental health. An observational research design with propensity score-matched samples compared the mental health of individuals who did (Nature Group Walkers, *n* = 1081) or did not (Non-Group Walkers, *n* = 435) attend nature group walks. A sub-sample of Frequent Nature Group Walkers (at least once per week, *n* = 631) was also investigated. Data were analyzed using multiple regression with an interaction term. All analyses were controlled for age, gender, and recent physical activity. Results showed that neither nature group walking, nor doing this frequently, moderated the effects of stressful life events on mental health. Using a main effects model, the positive associations of group walks in nature were at a greater magnitude than the negative associations of stressful life events on depression, positive affect, and mental well-being, suggesting an ‘undoing’ effect of nature group walks. Group walking schemes in natural environments may be an important public health promotion intervention for mental health.

## 1. Introduction

Mental illness is one of the most common non-communicable diseases in the United States [1] and Europe [2,3]. Currently, 20% of Americans [1,4] and about 27% of Europeans [5] experience a mental illness in a given year. The global economic burden of mental disorders is projected to be $6.0 trillion dollars in 2030—greater than the costs of cancer, diabetes, and respiratory disorders combined [6]. Prevention and low-cost amelioration of mental ill health is necessary in order to reduce healthcare demands and treatment costs [6]. 

The use of natural environments could be one such health promotion intervention [7,8,9,10,11,12,13]. A large body of empirical research has identified the role of natural environments in facilitating recovery from stress, e.g., [14,15], and treating depression, e.g., [16]. This suggests that nature could be an important resource for fostering resilience, by moderating the relationship between stressful life events and mental health. However, few studies have formally investigated this idea. 

National outdoor group walking programs could be a nature-based public health intervention to foster resilience. Walking is an accessible, low risk, and inexpensive form of physical exercise [17] that has been shown to reduce mental ill health [18,19]. As national group walk programs occur in outdoor environments [20], and given that both nature and walking help mental health, interventions such as this that combine nature exposure and walking may have additive beneficial effects for mental health. Indeed, systematic reviews and meta-analyses have demonstrated that walking in nature has added mental health benefits over walking in urban spaces [21] or indoors [21,22]. Group walks in natural environments have also been found to significantly improve mental health, compared to group walks in urban environments [23,24] or indoors [25]. This suggests that group walks in natural environments could be a nature-based therapy to help people cope with stressful life events. However, to date, no studies have investigated whether walking in nature can foster resilience [19,26]. This study specifically examines whether a national walking group program that occurs outdoors could be a socially prescribed nature-based public health intervention to foster resilience, by buffering the effects of recent stressful life events on mental health. In the following sections, we briefly review the literature on resilience, restorative environments, and the buffering effects of natural environments.

### 1.1. Resilience

Resilience is generally understood to be the ability to ‘bounce back’ from adversity [27]. Empirically, resilience is operationalized as the maintenance or recovery of well-being, following exposure to adversity or risk [28,29,30]. Adversity is the experience of life events or circumstances that threaten individual functioning or development [31], such as stressful life events (e.g., job loss, divorce, death of a family member) [32]. Stressful life events are a risk factor for mental illness, and are associated with higher depression and anxiety symptoms [33,34,35,36,37,38], perceived stress [39], and negative affect [40].

To facilitate resilience, protective factors are required [41]. Protective factors buffer the impacts of adversity, making the ability to ‘bounce back’ more likely [42,43]. Table 1 details the three levels at which protective factors occur [41,44,45]. Personal-level protective factors are attributes of the individual that facilitate the interpretation of, coping with, and reaction to adversity [30,41]. Family-level protective factors involve social support from family and friends. Community-level protective factors are the characteristics of the social and physical environment. Natural environments have been suggested as being potential community-level protective factors [31,45,46]. For example, wilderness camps may promote resilience, as the experience provides the opportunity to engage in activities and to develop new skills that develop feelings of self-esteem, competency [47], self-confidence, or self-efficacy [31]. Young people who participate in a wilderness program report significant improvements in self-concept, self-sufficiency, and self-esteem [47], as well as more positive emotions, relaxation, and less negative emotions and stress [48]. Natural environments may also foster post-adversity growth by promoting self-reflection [47,49,50], spiritual well-being [48,51,52], or the belief that life has meaning [46]. To date, no studies have investigated walking, or walking in nature, as a protective factor of resilience [19,26].

### 1.2. Restorative Environments

Two theories explain how natural environments can facilitate resilience: Stress Reduction Theory (SRT) and Attention Restoration Theory (ART). Both theories can be understood as “a complement to the study of stress and coping” [59] (p. 41). The SRT by Roger Ulrich posits that natural environments facilitate restoration and recovery from stress. Restorative outcomes of a nature experience include reduced physiological arousal, psychological stress, and negative affect, and enhanced positive affect [60,61]. With regard to resilience, the SRT has been tested to see whether nature can help with recovery from the after-effects of stress, as well as protect against future stressors. Exposure to nature after experiencing an acute stressor (e.g., a scary film) results in stress recovery [60,62,63]. However, the evidence is mixed for whether exposure to nature before experiencing an acute stressor can protect against subsequent stress responses [62,63]. Contact to nature has also been shown to facilitate restoration and stress recovery in samples experiencing chronic stressors. Access to nature at work facilitated stress recovery among male employees experiencing work-related stress [64]. The use of natural environments was found to improve mood and to reduce perceived stress and cortisol levels for individuals with poor mental health [65].

The ART by Rachel and Stephen Kaplan posits that natural environments contain stimuli that allow for the restoration of one’s ability to direct or focus attention [47]. Directed attention is an executive cognitive function, responsible for inhibitory control, and the ability to process information, plan, and solve problems [66,67]. The ability to direct attention is necessary for, among other things, fulfilling a task (e.g., writing a report), or for planning and managing behavior (e.g., achieving life goals) [66,67]. However, this cognitive capacity to direct attention is limited, and can become fatigued from continuous and prolonged use [47,67], or after sustained psychological or physiological stress responses to an adverse event [66]. Cognitive and behavioral consequences of directed attention fatigue include the inability to solve problems, or to cope with stress, impaired perception, impulsive behavior, and irritability with others [47,66,67,68]. The restoration of the ability to direct attention can occur through the use of a mode of attention that does not require any cognitive effort, called involuntary attention [47,66]. Environments with interesting stimuli will attract involuntary attention, which can enable the restoration of directed attention. Natural environments are theorized to be especially good settings for attention restoration, because they contain stimuli that attract involuntary attention [47,66,69].

According to the ART, there are two stages of a restorative experience [47,70]. The first stage is attentional recovery, which involves clearing one’s head of various thoughts and tasks, and the recovery of directed attention [47]. The second stage is reflection, which involves thinking about life matters, and reflecting on one’s life, goals, and priorities, and how to achieve them [47]. It is through this second stage of reflection that ART may help to facilitate resilience and post-adversity growth. Reflection is considered as being crucial for resilience; many resilience interventions train individuals in reflective cognitive practices [71,72]. Reflection enables one to “take stock” of the traumatic event and its impact [73] (p. 818), which is necessary for identifying, evaluating, and expressing post-adversity change [73]. The opportunity to think is a motivation for the use of local urban parks [51]. Wilderness experiences [47,74] and use of forests [75] and urban parks [49,50] have been shown to foster reflection. Natural environments can also foster reflection by enabling ‘peak experiences’ that give profound insights into a significant life issue, and understanding into one’s authentic self [76].

### 1.3. The Buffering Effect of Natural Environments

**Figure 1:**
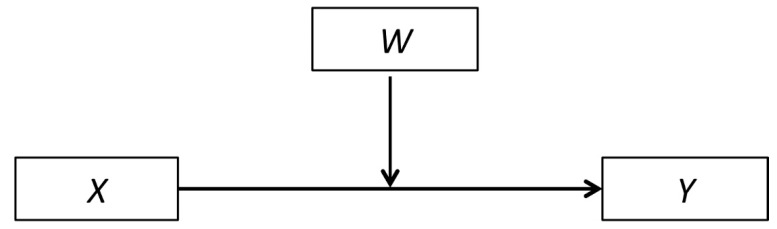
Moderation model where *X* represents an independent variable, *Y* the outcome variable, and *W* the moderating variable (figure based on [80]).

If natural environments are a protective factor for resilience, then nature should buffer against the negative health impacts of adversity [42,43,77]. Protective factors are statistically analyzed as being moderators [77,78]. A moderating variable changes the strength or direction of an association between independent and outcome variables [79]. As graphically depicted in Figure 1, a protective factor is a moderating variable (*W*) that buffers the effect of a negative independent variable (*X*) (e.g., adversity) on an outcome variable (*Y*) (e.g., mental health) [79,80], and this is measured using an interaction term, X*W (e.g., adversity*protective factor).

A few studies have explicitly examined natural environments as buffers from stressful life events. For example, the amount of nature in and around the home was found to moderate the effect of stressful life events on children’s psychological distress and global self-worth [81]. Stressful life events had less of an impact on children’s distress and self-worth when there was a high amount of nature near the home. Similarly, the amount of nature near a child’s school was also found to buffer the impact of stressful life events on a child’s perceived stress [82]. In adults, having a view of nature (i.e., trees, plants, foliage) from one’s office was found to moderate the negative impacts of job stress on one’s intention to quit their job—but not their general well-being [83]. van den Berg et al. [84] investigated whether the amount of green space 1 km and 3km from home would buffer the negative effects of recent stressful life events on physical and mental health. They found that the amount of green space within a 3 km radius around the home buffered the impact of recent stressful life events on physical health only. Adults who had experienced a stressful life event, and who lived within 3 km of a high amount of green space, reported fewer health complaints and better general health, than individuals who lived within 3 km of a low amount of green space. However, the amount of nature near the home—at either 1 km or 3 km—did not moderate the effects of stressful life events on mental health [84]. A limitation of van den Berg et al. [84] and these other studies [81,82,83] is that the actual use of natural environments by respondents is not known. As such, the actual use of nature as a buffer from stressful life events has not yet been investigated.

Other studies have investigated the protective effects of the actual use of natural environments for individuals experiencing stressful life events. However, because none of these studies formally tested for moderation, they do not provide evidence of the buffering effects of contact with nature. For example, a qualitative study with female victims of domestic violence found that gardening reduced feelings of stress, negative affect, and depression, and enhanced feelings of relaxation and empowerment [85]. A 12-week nature-based stress management course involving guided walks and gardening was found to significantly reduce burnout and stress-related health symptoms among individuals experiencing work-related stress [86]. For individuals who were highly affected by stressful life events, experiencing nature and walking in a natural environment were significantly and positively correlated with coping processes [87]. An intervention study of women with breast cancer found that women who engaged in nature-based activities showed significant improvement on directed attention, compared to women in the standard care control group [88]. An experimental study of university students experiencing academic exam stress found that students who walked in nature had significantly lower cortisol levels compared to students who watched nature indoors, although no significant difference was found between students in the walking in nature and the indoor treadmill condition [15]. However, students who walked in nature reported significantly greater positive affect, compared to students in both the indoor treadmill and indoor watching nature conditions [15]. A qualitative study identified that group walks in nature can help people to cope with stressful life events, such as bereavement [89]. Marselle et al. [90] found that group walks in nature were associated with better mental health, compared to non-group walkers, whilst recent stressful life events were associated with poorer mental health. This suggests a possible protective effect of group walks in nature from recent stressful life events, although a moderation analysis was not conducted [90]. As these studies vary in the quality of their research designs, populations, and sample sizes, there are inherent risks of bias that could make comparisons difficult.

### 1.4. Frequency of Visiting a Natural Environment for a Buffering Effect

Recent discussion within the nature-health literature has examined the issue of ‘dose’, or the amount of contact with nature that is needed to foster a health benefit [91]. A “dose of nature” is conceptualized to include the magnitude, frequency, and duration of exposure to nature, the type and qualities of the natural environment, as well as the type of contact with and experience of nature [8]. Previous research suggests that frequency of nature interactions may be important for fostering resilience. For example, female victims of domestic violence who spent six hours or more per week gardening experienced more therapeutic and positive feelings than women who spent three hours or less per week gardening [85]. Ottosson and Grahn [87] found that the negative effects of stressful life events on attention and mental health were weaker for individuals who had many experiences of nature. The authors conclude that: “access to nature in everyday life seems to have a buffering effect on people’s mental state… If people in crisis have *many* nature experiences, they tend to experience an improved state of health” (emphasis added) [87] (p. 66). From this research, it appears that frequent nature contact may be important for fostering resilience.

### 1.5. The Present Study

The aim of the present study was to investigate whether nature-based group walks could foster resilience by buffering the adverse effects of stressful life events on mental health. Aspects of this intervention that may contribute to this buffering effect on mental health could be the natural environment, walking itself, and the social interaction of the group. All three of these elements were included in our study design and analyses. This study builds upon previous research in three ways: it formally tests whether walking in nature is a protective factor that can foster resilience [19,26,90]; it addresses the limitations of van den Berg et al. [84] by assessing actual use of green space as a buffer of stressful life events; and it examines whether frequent nature experiences are required for resilience, as suggested by previous literature [85,87].

**Figure 2:**
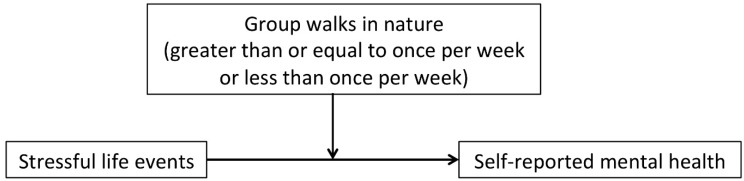
Conceptual model for the analysis of group walks in nature as a moderator of the relationship between stressful life events and mental health (based on [84]).

Figure 2 illustrates the conceptual model for the analysis of our study; group walks in nature interact with stressful life events to buffer the adverse effect of such events on self-reported mental health. Our main hypothesis was that the adverse impacts of experiencing stressful life events on mental health would be less severe for individuals who take part in group walks in nature. Our second hypothesis was that the buffering effects would be stronger for those who walk frequently (at least once a week) in nature with a group.

## 2. Methods

### 2.1. Study Design and Participants

Participants were recruited from an observational study investigating the wellbeing benefits of Walking for Health (WfH) health walks [90]. WfH is an England-wide network of health walk schemes that regularly provide free, short, led group walks that occur at a moderate pace, and that are open to all [92]. With 1800 weekly health walks attended by an average of 24,000 walkers [93], WfH is one of the largest public health interventions for physical activity in the UK [94]. WfH walks occur in a variety of outdoor environments [20], most of which take place in a setting within nature (e.g., urban park, countryside park) [24].

The participant inclusion criteria are listed in Table 2. Nature Group Walkers were defined as regular WfH attendees who took part in at least one WfH walk in a natural environment over the previous three months. Additional eligibility criterion for Group Walkers was that the main type of environment for one’s WfH walks was in a natural environment (i.e., natural and semi-natural places, farmland, green corridor, coastal area, urban green space, or any mixture of the above) [24]. The subsample of Frequent Nature Group Walkers was defined as attending a WfH walk in the natural environment at least once per week in the previous three months. The Comparison Group was defined as individuals who had not taken part in any group walk (WfH or otherwise) in the previous nine months. All participants were recruited from a sampling frame, provided by WfH, of all individuals who had attended at least one WfH walk, provided an email address, and had gave their consent to be contacted for evaluations.

Online questionnaires were used to collect data at Time 1 (T1) (baseline mental health), and three months later at Time 2 (T2) (stressful life events, mental health outcomes and covariates). Additional health, past physical activity, and demographic data for all participants was obtained at the participant’s first WfH walk (T0). All participants gave informed consent, were resident in England, and aged 18 or older. Ethical approval was obtained from De Montfort University’s Human Research Ethics committee. For more information about participant recruitment and research design, see Marselle et al. [90].

### 2.2. Measures

#### 2.2.1. Mental Health Indicators

1. Perceived stress. The 10-item Perceived Stress Scale [95] assessed how frequently participants experienced certain stressful thoughts and feelings (e.g., “How often have you felt nervous or ‘stressed’? How often have you felt that you were on top of things”) in the past month, on a 5-point scale (0 = never; 4 = very often). Total scores range from 0 to 40; higher scores indicate greater psychological stress.
2. Depression. The 10-item Major Depressive Inventory [96] assessed how frequently participants experienced symptoms of depression (e.g., “Have you lost interest in daily activities? Have you had trouble sleeping at night?”) in the past two weeks, on a 6-point scale (0 = at no time; 5 = all the time). Total scores range from 0 (no depression) to 50 (extreme depression) [96].
3. Negative and positive affect. The Positive and Negative Affect Schedule (PANAS) [97] assessed both negative and positive affect. Participants rated the frequency of experiencing 10 negative (e.g., upset, guilty) and 10 positive (e.g., interested, excited) emotions in the past two weeks, on a 5-point scale (1 = very slightly or not at all; 5 = extremely). For each subscale, total scores range from 10 to 50; higher scores demonstrate greater negative or positive affect.
4. Mental well-being. Participants rated statements on the 14-item Warwick Edinburgh Mental Well-being Scale [98] in relation to their experiences (e.g., “I’ve been feeling optimistic about the future; “I’ve been feeling useful”) during the past two weeks, on a 5-point scale (1 = none of the time; 5 = all of the time). The resulting scores range from 14 to 70; higher scores indicate higher levels of mental well-being.

#### 2.2.2. Stressful Life Events

Stressful life events have been investigated in previous studies of resilience [30,99,100]. The List of Threatening Experiences questionnaire (LTE-Q) [101,102] is a self-report questionnaire to determine the occurrence of experiencing stressful life events. Participants responded to whether they had or had not experienced each of the following 11 stressful life events in the previous three months: serious illness, injury or assault to self, serious illness, injury or assault to a close relative, death of a close relative, death of a family member or other relative, marital separation or relationship breakup, interpersonal problems, unemployment, unsuccessfully looking for work for more than one month, major financial crisis, legal problems, or property loss [103]. The LTE-Q was used by van den Berg et al. [84]. Following van den Berg et al. [84], these data were dichotomized, to assess whether or not a person experienced one or more stressful life events over the previous three months (0 = No recent stressful life events, 1 = one or more recent stressful life events).

#### 2.2.3. Group Walks in Nature

The hypothesized moderator of the effect of stressful life events in the main sample analyses was nature group walk participation (0 = Comparison Group, 1 = Nature Group Walkers). The moderator in the subsample analyses was frequent nature group walk participation (0 = Comparison Group, 1 = Frequent Nature Group Walkers).

#### 2.2.4. Covariates

To address the possibility that it is the walking itself and or the social interaction within a nature-based group walk that buffers the effects of stressful life events, recent physical activity and social well-being were assessed. Additional covariates known to influence mental health were also assessed. All covariates were measured at T2, except for demographic and health data, which were measured at T0.

1. Recent physical activity is associated with emotional resilience [104]. The participants’ recent physical activity was assessed with a single item that asked about the number of days engaging in 30 minutes of physical activity in the previous week [105]. Responses were recorded on an 8-point scale (0 = 0 days; 7 = 7 days).
2. Social well-being has been shown to buffer the effect of stressful life events on health [106], depression [107], and negative affect [40]. To assess whether participants in the two conditions differed on social well-being, the 10-item Appraisal subscale of the Interpersonal Support Evaluation List [108] was used. The scale measured the perceived availability of emotional social support (e.g., “There are several people that I trust to help solve my problems; I feel there is no one I can share my most private worries and fears with”). Participants rated how true each statement was, on a 4-point scale (0 = definitely false; 3 = definitely true). Higher scores indicated greater emotional social support.
3. Connection to nature is associated with emotional well-being [109] and positive affect [110], and it may contribute to group walkers’ mental well-being [111]. To assess group differences, the Connectedness to Nature scale was used [112]. This 14-item scale assesses participants’ emotional and experiential connections to the natural world (e.g., “I often feel a sense of oneness with the natural world around me; I often feel disconnected from nature”). Participants are asked to rate how much they agree with each statement on a 5-point scale (1 = strongly disagree; 5 = strongly agree). Higher scores reflect greater feelings about one’s connection to nature.
4. Psychological resiliency is a personal-level protective factor that buffers against the development of mental illness following adversity [53,54]. It is positively associated with positive mental health [113,114,115,116,117,118], and correlated with time spent in nature [119]. To assess group differences on psychological resiliency, the 10-item Connor-Davidson Resilience Scale (CD-RISC) [120,121] was used (e.g., “I am able to adapt when changes occur; I tend to bounce back after illness, injury or other hardships”). Participants were asked to rate how much they agreed with each statement on a 5-point scale (0 = not true at all; 4 = true nearly all of the time). Higher scores indicate greater psychological resiliency.
5. Demographic and health data of participants (measured at T0) included: age, gender, ethnicity, marital status, highest level of education, and social deprivation [122], as well as whether the participant was referred to WfH by their general practitioner (GP), health screening conditions that may affect walking group participation (e.g., pains in the chest when exercising, joint pain), diagnosed medical condition (e.g., diabetes, heart disease), disability (e.g., physical, sensory), and past physical activity [123].

### 2.3. Statistical Analysis

#### 2.3.1. Propensity Score Matching

Due to the observational research design, there were significant differences in demographics, health, and past stressful life events between the main sample groups (Nature Group Walkers and Comparison Group) [90], as well as between the subsample groups (Frequent Nature Group Walkers and Comparison Group). In order to reduce the effect of these confounding variables on the analyses, propensity score matching (PSM) [124] was used to make the groups comparable. PSM is recommended to reduce bias in natural experiments [125], and it has been used in public health research investigations of outdoor physical exercise [126,127,128]. For both samples, propensity scores were estimated by using logistic regression, with group walk participation as the outcome variable; predictors included variables known to affect both participation in a nature-based walking program, and the mental health indicators (i.e., age, gender, marital status, ethnicity, area deprivation, education, health condition, existing medical conditions, disability, past physical activity, and past stressful life events) [129,130,131]. Excluded from the PSM are all mental health indicators, and any covariates that are affected by participation in a nature-based walking program (i.e., recent physical activity, social support, connection to nature, and resilience) [129,130,131]. Participants were matched using 1:1 nearest-neighbor matching with replacement, as recommended when there are fewer “control” than “treated” participants [132]. The PSM sample was assessed statistically, numerically, and graphically, to ensure that the two groups were similar for the selected covariates after matching. The PSM procedure was performed using the SPSS PSM plug-in “psmatching” [133].

#### 2.3.2. Analysis

All analyses were performed using IBM SPSS 22.0 (IBM, Armonk, NY, USA), and they were weighted by the propensity score weight. Chi-square and independent sample *t*-tests evaluated group differences in demographics and health. Group differences on recent physical activity, social support, connection to nature, and psychological resiliency were also assessed with independent sample *t*-tests. Hierarchical multiple regression was used for all buffering analyses; ANCOVA could not be used, as SPSS rounds propensity score sampling weights to the nearest whole number, thus omitting the sampling weights [134,135,136]. Recent stressful life events and group walk participation were both centered around the mean, in order to reduce multicollinearity with the interaction term [137]. To test whether group walks in nature facilitate resilience, an interaction term was computed by multiplying the mean-centered recent stressful life events variable by the mean-centered nature group walk participation variable (i.e., centered recent stressful life events × centered nature group walk participation) [138,139,140]. If the interaction term is significant, then moderation is considered to be present [79], and nature group walks would be considered to buffer the impact of stressful life events. In all regression analyses, covariates (age, gender, T1 mental health, and recent physical activity) were entered in the first step. Recent stressful life events, nature group walk participation, and the interaction term were entered in the second step with the enter method. Regression analyses were run separately for each mental health indicator. There was no evidence of multicollinearity, and Variance Inflation Factor (VIF) and Tolerance were within limits [141]. Residuals were normally distributed, except for depression and negative affect. Because depression and negative affect were positively skewed, we applied a log-transformation to improve the normal distribution of the residuals. However, untransformed data were reported when the results for the untransformed data were similar to the transformed data. Listwise deletion was applied, and significant levels were set at *p* < 0.05.

## 3. Results

### 3.1. Participants

The main study sample, after PSM, comprised 1516 participants (*n* = 1081 Nature Group Walkers, *n* = 435 Comparison Group). The subsample, after PSM, consisted of 937 participants (*n* = 631 Frequent Nature Group Walkers, *n* = 306 Comparison Group). In the main sample, after matching, the only significant difference between the two groups was for health conditions (Supplementary Table S1). A greater proportion of the Comparison Group had a health condition (20.5%) than the Nature Group Walkers (16.2%), *X*^2^(1) = 4.04, *p* = 0.04; this was subsequently included in the analysis as a covariate. There were no significant differences between the two groups in the subsample after PSM (Supplementary Table S2). Both samples (main and subsample) were predominately 55 years or older, female, of white ethnicity, in a relationship, university-educated and had lived in the least socially deprived neighborhoods (Supplementary Tables S1 and S2).

### 3.2. Group Differences for Social Well-Being, Recent Physical Activity, Connectedness to Nature, and Psychological Resiliency

There were no significant differences for social well-being in both the main sample (*p* = 0.74) and the Frequent Nature Group Walkers subsample (*p* = 0.31) (Supplementary Table S3). Significant group differences on recent physical activity were found for both the main sample (*p* < 0.001) and the Frequent Nature Group Walkers subsample (*p* < 0.001) (Supplementary Table S3). Nature Group Walkers and Frequent Nature Group Walkers were engaged in more physical activity in the previous week, in contrast to the Comparison Groups. For the main sample, there was a significant group difference for connection to nature (*p* = 0.04), with a marginally significant difference in psychological resiliency (*p* = 0.05) (Supplementary Table S3). Consequently, recent physical activity, connection to nature, and psychological resiliency were included as covariates in the regression for the main sample. For the Frequent Nature Group Walkers subsample, there was a significant group difference for psychological resiliency (*p* = 0.04), with no significant difference for connection to nature (*p* = 0.63) (Supplementary Table S3). Recent physical activity and psychological resiliency were included as covariates in the analyses for the Frequent Group Walkers subsample.

### 3.3. Frequency of Stressful Life Events Experienced by the Group

For the main sample, there was no significant difference in the experience of recent stressful life events between the two groups (*p* = 0.68) (Supplementary Table S4). For the subsample, the Comparison Group experienced more recent stressful life events (*M* = 0.48, *SD* = 0.50), than for the Frequent Nature Group Walkers (*M* = 0.41, *SD* = 0.49). This difference was marginally significant, *t*(595.27) = 2.01, *p* = 0.05 (Supplementary Table S5).

### 3.4. Buffering Effects of Nature Group Walks—Main Hypothesis

**Figure 3:**
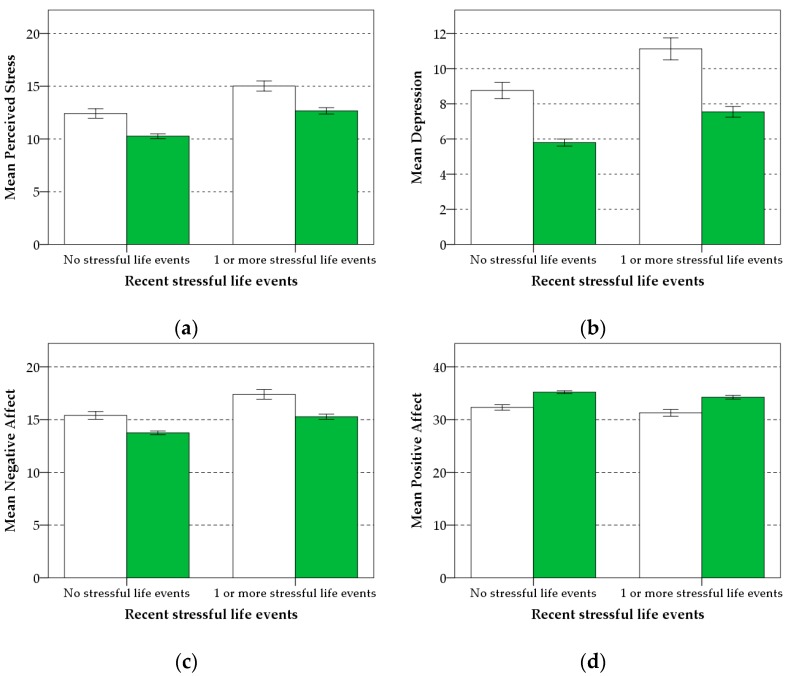

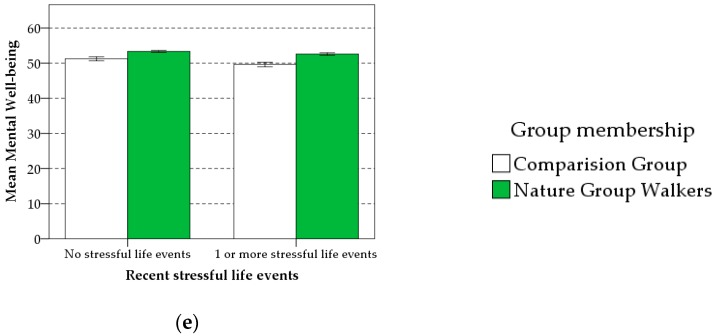
Mean perceived stress (**a**), depression (**b**), negative affect (**c**), positive affect (**d**), and mental well-being (**e**) as a function of recent stressful life events and nature group walk participation. Main sample (*n* = 1506). Error bars are ±1 SE of the mean.

There was no significant interaction of group walks in nature with recent stressful life events for all five mental health indicators (see Table 3). The impact of stressful life events on mental health was similar for both groups (see Figure 3). As there was no evidence of moderation, a parsimonious (main effects) model, without the interaction term, was analyzed [142]. Both recent stressful life events and nature group walks had significant influences on all mental health indicators. Experiencing at least one recent stressful life event was significantly associated with an increase in perceived stress, depression, and negative affect, and a reduction in positive affect and mental well-being (see Table 4). In contrast, participation in nature group walks was significantly associated with a reduction in perceived stress, depression, and negative affect, and an increase in positive affect and mental well-being (see Table 4). The standardized regression coefficients for nature group walks on depression (*β* = −0.116), positive affect (*β* = 0.108), and mental well-being (*β* = 0.68) were higher and in the opposite direction to the standardized regression coefficients for recent stressful life events on depression (*β* = 0.104), positive affect (*β* = −0.057), and mental well-being (*β* = −0.041), suggesting an ‘undoing’ effect of nature group walks.

### 3.5. Buffering Effects of Frequent Nature Group Walks—Second Hypothesis

**Figure 4:**
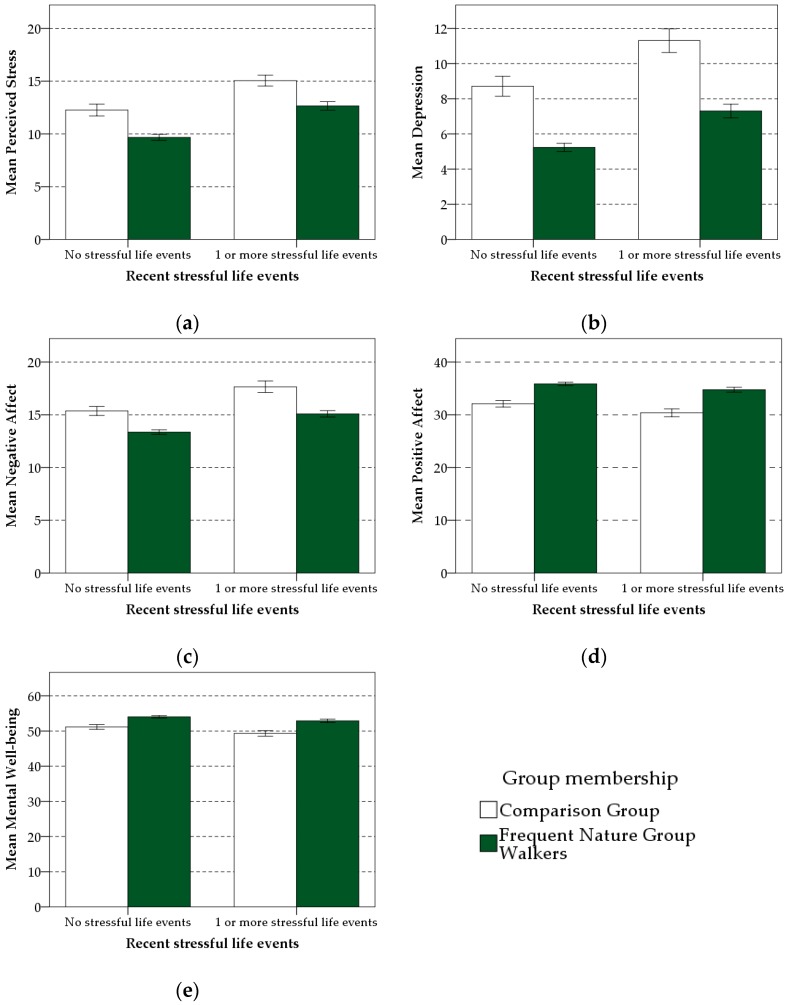
Mean perceived stress (**a**), depression (**b**), negative affect (**c**), positive affect (**d**), and mental well-being (**e**) as a function of recent stressful life events and frequent nature group walk participation. The frequent group walkers subsample consists of individuals from the main sample who attended a group walk in the natural environment at least once per week (*n* = 930). Error bars are ±1 SE of the mean.

There was no significant interaction of frequent group walks in nature with recent stressful life events for all five mental health indicators (see Table 5). The impact of recent stressful life events on mental health was the same for both the Frequent Nature Group Walkers and the Comparison Group (see Figure 4). As there was no evidence of moderation, a parsimonious (main effects) model, without the interaction term, was analyzed [142]. Both recent stressful life events and frequent nature group walks had a significant influence on perceived stress, depression, negative affect, and positive affect. Experiencing at least one recent stressful life event was associated with an increase in perceived stress, depression, and negative affect, and a reduction in positive affect (see Table 6) whereas frequent participation in nature group walks was associated with a reduction in perceived stress, depression, and negative affect, and an increase in positive affect (see Table 6). The standardized regression coefficient for frequent nature group walks on depression symptoms (*β* = −0.115) and positive affect (*β* = 0.148) was higher, and in the opposite direction, compared to the regression coefficient for recent stressful life events on depression (*β* = 0.096) and positive affect (*β* = −0.054), suggesting an ‘un-doing’ effect by frequent group walks in nature. Recent stressful life events had a marginally significant negative influence on mental well-being (*β* = −0.040, *p* = 0.06), whilst frequent group walks in nature had a significant influence on mental well-being in the opposite direction (*β* = 0.089, *p* < 0.001).

## 4. Discussion

We explored whether a national group walking scheme could be a nature-based therapy to foster resilience in the general population. Specifically, we investigated whether the actual use of nature through group walks—and frequent group walks—would buffer the impact of recent stressful life events on five self-reported mental health indicators: perceived stress, depression, negative affect, positive affect, and mental well-being. There was no evidence for such a buffering effect. The impact of recent stressful life events on all mental health indicators was the same for the Comparison Group as it was for both Nature Group Walkers and Frequent Nature Group Walkers. This result fits with van den Berg et al.’s [84], study which found that the amount of green space near the home did not buffer the effect of recent stressful life events on mental health.

Main effects were, however, found for both recent stressful life events and nature group walks. Experiencing at least one stressful life event was associated with an increase in perceived stress, depression, and negative affect, and a decrease in positive affect and mental well-being. Nature group walks—and frequent nature group walking—were associated with a reduction in perceived stress, depression, and negative affect, and an increase in positive affect and mental well-being. Similar main effect results were also found in Marselle et al. [90]. Contrary to previous literature [85,87], frequent nature-based group walks did not foster resilience. This pure main effect model suggests that—irrespective of whether the persons are experiencing stressful events—any group walks in nature, regardless of frequency, have a beneficial effect on mental health [143]. In other words, walking with others in nature is beneficial to mental health, but it may not help to buffer the impact of recent stressful life events.

In both samples, the main effect results suggest that nature group walks can ‘un-do’ or reduce the effects of recent stressful life events on depression and positive affect. The positive association of nature group walks and frequent nature group walks on these two aspects of mental health was at a greater magnitude than the negative association of recent stressful life events. Wardenaar et al. [38] found an ‘un-doing’ effect in their assessment of the effects of positive life events on depression; positive life events were associated with a significant decrease in depression at an equal or greater magnitude than negative life events. Similarly, Childs and de Wit [104] found that people who did regular exercise showed less of a decline in positive affect when exposed to a stressor than non-exercisers, suggesting that regular exercisers may be more resistant to the effects of stress on positive affect. Our ‘un-doing’ result suggests that nature group walks may dampen the impact of stressful events on mental health. To use a metaphor, nature group walks reduce the impact of a bullet, but they do not provide bulletproof protection. Although, group walks in nature may not be a ‘magic bullet’ for adult stressful life events—they could be a “stepping stone to recovery” [144] (p. 9).

The Stress Reduction Theory (SRT) provides a useful explanation for the ‘un-doing’ effect found in this study. According to SRT, natural environments help to facilitate recovery from the after-effects of stress [60]. Results from laboratory studies have shown that exposure to nature after experiencing an acute stressor results in physiological stress recovery [60,62,63], yet the evidence is mixed for whether exposure to nature before experiencing an acute stressor can protect against subsequent physiological stress responses [62,63]. Our study suggests that individuals who take part in nature-based group walks exhibit recovery from the after-effects of at least one recent stressful event on mental health.

A possible explanation for the absence of a buffering effect could be the social context of the group walks themselves. People prefer being alone in nature when they are in need of attentional recovery [145], and when they are experiencing emotional or cognitive stress [146]. Part of the reason for why nature can be restorative is the absence of social feedback [146,147]. This is because social interaction can make demands on directed attention [148], and pull one’s attention away from the natural environment [146,149]. As such, the presence of others could be a constraint to restoration [148], which could hinder the opportunity of natural environments to foster resilience. In other words, walking and talking with others while out in nature could mean that one does not have the opportunity to regain the capacity to direct attention and to engage in reflection—psychological experiences which may facilitate resilience. Indeed, previous research has found that the restorative effects of natural environments are diminished when one is with other people [150,151,152], and that being with others in the natural environment negatively influences the likelihood of reflection [148].

Does this mean one needs to be alone in nature for there to be a buffering effect of stressful life events on mental health? Unfortunately, neither the SRT nor the ART addresses the influence of social context on psychological restoration [148]. Solo experiences in wilderness result in reflection [47,75,76], critical self-examination, and reconnection to one’s true self [76], although, this may only occur when one feels safe in the natural environment. Staats and Hartig [148] found that solitude enhanced both attentional recovery and reflection, but only when safety was not a concern. When safety was a concern, being with others in a natural environment enabled attentional recovery and reflection by providing feelings of safety [148]. Yet, positive mental health and well-being have been found in people who walk with others in nature [18,23,90,92,153,154,155,156] suggesting that people may be experiencing attentional recovery and reflection, even when walking with others in nature. This is yet to be investigated. Future studies investigating the buffering effects of nature-based activities should consider the social context.

### 4.1. Strengths, Limitations, and Future Directions

This study makes an important contribution to the literature on the use of nature-based group walks as a public health intervention to foster resilience, as no studies, to date, have investigated walking as a protective factor of resilience [19,26]. Use of the PSM method improved the ability to investigate the effect of participation in a national outdoor group walk program on mental health. The large sample of adults from the general population of England, engaged in a national walking program, enabled the statistical control of other significant predictors of resilience and well-being, and sufficient power to detect a small yet significant effect. The actual use of nature on mental health was measured, as opposed to the presence of green space near the home [84] or images of nature [62].

While the PSM method ensured that there were no significant group differences on measured covariates, it remains possible that differences existed for unmeasured confounding variables [157]. Although we controlled for the effects of other predictors of well-being and resilience in the regression model, other explanatory variables could account for group differences. Due to the observational research design, we are unable to state causality. It could be that individuals who experience better mental health may be more likely to take part in group walks in nature. Individuals in the Comparison Group may have other nature exposure opportunities beyond a walking group, which were not measured in this analysis. This unmeasured exposure to nature could also account for the non-significant buffering effects found here. As such, future studies of the buffering effect of nature could investigate all types of nature exposure. While physical activity was controlled for, the current research design could not separate the effects of the natural environment from the effects of walking. Future exploration of the buffering effects of nature-based group walks could include a comparison group that takes part in group walks indoors [25], or in an urban environment [23]. Nature group walkers were already participating in WfH at the start of this study; thus no measurement of mental health before the commencement of participation in the nature group walks exists. Longitudinal research is needed with new group walkers, to examine the buffering effect of nature group walks. The study took place over the changing seasons; as such, the data may reflect a seasonal effect. However, walking in a forest in the winter when trees are without leaves has been found to have positive, restorative effects on well-being [158]. The reliance on self-reported measures may mean that the estimates of association were inflated; future studies may wish to use physiological measures. Finally, participants were mostly female, older, white, and affluent; while they are likely to be unrepresentative of the adult general population living in England, participants were representative of the population involved in WfH [94].

Future studies of the buffering effect of the actual use of nature could investigate the effects of nature-based group walks compared to nature-based walks alone. Activities that involve intentional, more active participation in nature [159], such as horticultural therapy, forestry therapy, or wilderness therapy, could be formally investigated, to determine whether they foster resilience. Future research could also investigate whether the actual use of nature could buffer stressful life events by reducing rumination—prolonged, maladaptive attentional focus on the causes and consequences of one’s emotions [160,161]. Stopping rumination is important for facilitating recovery from stressful life events, and preventing mental illness [162]. While walking alone in nature [160,161] and nature-based group activities like horticultural therapy [162] and group walks in nature [144,153] were found to reduce rumination, no study has investigated resilience in this context. As psychological resiliency is often used as an outcome measure in interventions to enhance resilience [26], and resilience is correlated with time spent in nature [119], future studies may wish to investigate whether group nature walks can increase psychological resiliency.

### 4.2. Implications

Organized group walks enable individuals to visit a natural environment that they may not have gone to on their own [163,164], thereby enhancing their likelihood of restoration [148]. Whilst the restorative benefits of being in nature with others might be reduced (compared to being alone) [152], the results in the present study show there is still a mental health benefit. In this way, national group walking schemes could be a non-medical community activity to which GPs could provide social prescriptions [165,166]. Social prescribing to nature-based programs, such as group walks in nature, gives local commissioners of health services the opportunity to bring people into contact with nature for mental health benefits [167].

## 5. Conclusions

This comparison study investigated whether the actual use of nature through group walks could be used as a nature-based therapy to foster resilience in the general population. No evidence was found for a buffering or protective effect of group walks in nature from recent stressful life events on mental health. Main effects were, however, found for both recent stressful life events and nature group walks. Further, group walks in nature appear to ‘un-do’ or dampen the effects of at least one stressful life event on depression, positive affect and mental well-being. This suggests that group walking schemes in natural environments may be an important public health promotion intervention for mental health.

## Figures and Tables

**Table 1:** Protective factors that facilitate resilience, occurring at the individual, family, and community levels.

Level of Protective Factor	Examples
Individual	Psychological resiliency [53,54] Age [30,55,56] Education [30,55,56]
Family	Social support from family or friends [41,45,57]
Community	Living in an affluent neighborhood [58] Public safety [31] Natural environments [31,45,46]

**Table 2:** Inclusion criteria for participant groups in the current study.

Group	Inclusion Criteria
Nature Group Walkers	Attend at least one WfH group walk in the six months prior to T1 Attended at least one WfH walk in the 3-month interim between T1 and T2 Main type of environment for WfH walks was the natural environment (i.e., natural and semi-natural places, green corridor, farmland, urban green space, coastal area, or a mixture of any of the above) between T1 and T2
Frequent Nature Group Walkers	Same inclusion criteria as Nature Group Walkers above, and Attended a WfH group walk in the natural environment at least once per week in the 3-month interim between T1 and T2
Comparison Group	Did not attend any group walk (WfH or otherwise) in the six months prior to T1, nor in the 3-month interim between T1 and T2

Note. WfH = Walking for Health; T1= Time 1; T2 = Time 2.

**Table 3:** Moderation results assessing group walks in nature, recent stressful life events, and the interaction term on perceived stress, depression, negative affect, positive affect, and mental wellbeing. Main sample (*n* = 1506 ^a^).

	Perceived Stress			Depression ^1^			Negative Affect ^2^			Positive Affect			Mental Well-Being		
Final Step	*B*	*SE*	*β*	*B*	*SE*	*β*	*B*	*SE*	*β*	*B*	*SE*	*β*	*B*	*SE*	*β*
Baseline outcome	0.49	0.02	0.46 ***	0.02	0.001	0.43 ***	0.52	0.02	0.52 ***	0.43	0.02	0.42 ***	0.48	0.02	0.50 ***
Recent physical activity ^b^	−0.05	0.06	−0.01	−0.01	0.003	−0.06 **	−0.08	0.05	−0.03	0.44	0.07	0.11 ***	0.24	0.07	0.06 **
Resiliency	−0.32	0.02	−0.31 ***	−0.01	0.001	−0.22 ***	−0.16	0.02	−0.20 ***	0.36	0.03	0.30 ***	0.35	0.03	0.28 ***
Connectedness to nature	−0.02	0.02	−0.02	−0.001	0.001	−0.02	−0.02	0.01	−0.03	0.09	0.02	0.10 ***	0.05	0.02	0.05 **
Recent stressful life event ^c^	1.73	0.23	0.13 ***	0.07	0.01	0.11 ***	1.20	0.20	0.11 ***	−0.86	0.27	−0.06 ***	−0.66	0.27	−0.04 **
Group walks in nature ^d^	−1.14	0.26	−0.08 ***	−0.08	0.02	−0.11 ***	−0.62	0.23	−0.05 **	1.78	0.30	0.11 ***	1.17	0.30	0.07 ***
Recent stressful life event × group walks in nature	−0.28	0.51	−0.01	0.02	0.03	0.02	−0.04	0.45	−0.002	−0.04	0.59	−0.001	0.66	0.60	0.02
R^2^ for Step 1	0.52 ***			0.38 ***			0.44 ***			0.51 ***			0.56 ***		
Change R^2^	0.02 ***			0.02 ***			0.02 ***			0.02 ***			0.01 ***		

Note: The final step is shown. In all regression analyses, baseline outcome, recent physical activity, resiliency, and connectedness to nature were entered in the first step. Recent stressful life events, group walks in nature, and the interaction term were entered in the second step with the enter method. All analyses were controlled for age, gender, and health condition, prior to the first WfH walk. Recent stressful life events and group walks in nature were centered at their means. Higher scores indicated greater perceived stress, depression, negative affect, positive affect, or mental well-being. ^a^ Propensity-matched sample; analysis weighted by propensity score weight. ^b^ Physical activity in the previous week. ^c^ Recent stressful life events experienced in the previous three months: 0 = No stressful life events, 1 = One or more stressful life events. ^d^ Group walk participation: 0 = Non-group Walkers, 1 = Nature Group Walkers. ^1^ Log-transformed data. ^2^ Untransformed results are reported, as results for the untransformed data were very similar to the log-transformed data. *B*, standardized coefficient; SE, standard error; β, standardized coefficient. ** *p* < 0.01. *** *p* < 0.001.

**Table 4:** A main effects model assessing the effect of group walks in nature and recent stressful life events on perceived stress, depression, negative affect, positive affect, and mental well-being. Main sample (*n* = 1506 ^a^).

	Perceived Stress			Depression ^1^			Negative Affect ^2^			Positive Affect			Mental Well-Being		
Final Step	*B*	*SE*	*β*	*B*	*SE*	*β*	*B*	*SE*	*β*	*B*	*SE*	*β*	*B*	*SE*	*β*
Baseline outcome	0.49	0.02	0.46 ***	0.48	0.02	0.50 ***	0.52	0.02	0.52 ***	0.43	0.02	0.42 ***	0.48	0.02	0.50 ***
Recent physical activity ^b^	−0.05	0.06	−0.01	−0.01	0.003	−0.06 **	−0.08	0.05	−0.03	0.44	0.07	0.11 ***	0.24	0.07	0.06 **
Resiliency	−0.32	0.02	−0.31 ***	−0.01	0.001	−0.20 ***	−0.16	0.02	−0.20 ***	0.36	0.03	0.30 ***	0.35	0.03	0.28 ***
Connectedness to nature	−0.02	0.02	−0.02	−0.001	0.001	−0.04	−0.02	0.01	−0.03	0.09	0.02	0.09 ***	0.05	0.02	0.05 **
Recent stressful life event ^c^	1.73	0.23	0.13 ***	0.07	0.01	0.10 ***	1.20	0.20	0.11 ***	−0.86	0.27	−0.06 ***	−0.66	0.27	−0.04 **
Group walks in nature ^d^	−1.14	0.26	−0.08 ***	−0.08	0.02	−0.12 ***	−0.62	0.23	−0.05 **	1.78	0.30	0.11 ***	1.17	0.30	0.07 ***
R^2^ for Step 1	0.52 ***			0.44 ***			0.44 ***			0.51 ***			0.56 ***		
Change R^2^	0.02 ***			0.02 ***			0.02 ***			0.02 ***			0.01 ***		

Note. Final step shown. In all regression analyses, baseline outcome, recent physical activity, resiliency, and connectedness to nature were entered in the first step. Recent stressful life events and group walks in nature were entered in the second step with the enter method. All analyses controlled for age, gender, and health condition prior to the first WfH walk. Higher scores indicate greater perceived stress, depression, negative affect, positive affect, or mental well-being. ^a^ Propensity-matched sample; analysis weighted by the propensity score weight. ^b^ Physical activity in the previous week. ^c^ Recent stressful life events experienced in the previous 3 months: 0 = No stressful life events, 1 = One or more stressful life events. ^d^ Group walk participation: 0 = Non-group Walkers, 1 = Nature Group Walkers. ^1^ Log-transformed data. ^2^ Untransformed results are reported, as results for the untransformed data were similar to the log-transformed data. *B*, standardized coefficient; SE, standard error; β, standardized coefficient. ** *p* < 0.01. *** *p* < 0.001.

**Table 5:** Moderation results assessing frequent group walks in nature, recent stressful life events, and the interaction term on perceived stress, depression, negative affect, positive affect, and mental well-being. Frequent Nature Group Walkers subsample ^a^ (*n* = 930 ^b^).

Final Step	Perceived Stress			Depression ^1^			Negative Affect ^1^			Positive Affect			Mental Well-Being		
	*B*	*SE*	*β*	*B*	*SE*	*β*	*B*	*SE*	*β*	*B*	*SE*	*β*	*B*	*SE*	*β*
Baseline outcome	0.44	0.03	0.42 ***	0.51	0.03	0.53 ***	0.51	0.03	0.54 ***	0.53	0.03	0.50 ***	0.48	0.03	0.49 ***
Recent physical activity ^c^	−0.10	0.08	−0.03	−0.22	0.08	−0.06 **	−0.11	0.07	−0.04	0.40	0.09	0.10 ***	0.22	0.09	0.05 *
Resiliency	−0.34	0.03	−0.34 ***	−0.20	0.03	−0.20 ***	−0.17	0.02	−0.20 ***	0.31	0.03	0.25 ***	0.39	0.04	0.31 ***
Recent stressful life events ^d^	1.86	0.30	0.14 ***	1.27	0.31	0.10 ***	1.20	0.25	0.12 ***	−0.84	0.34	−0.05 *	−0.65	0.35	−0.04
Frequent group walks in nature ^e^	−1.26	0.32	−0.09 ***	−1.60	0.34	−0.12 ***	−0.71	0.27	−0.06 **	2.40	0.37	0.15 ***	1.51	0.38	0.09 ***
Recent stressful life events × Frequent group walks in nature	−0.20	0.62	−0.01	−0.26	0.65	−0.01	−0.25	0.52	−0.01	0.94	0.73	0.03	0.84	0.74	0.02
R^2^ for Step 1	0.52 ***			0.48 ***			0.49 ***			0.53 ***			0.57 ***		
Change R^2^	0.03 ***			0.02 ***			0.02 ***			0.03 ***			0.01 ***		

Note. Final step shown. In all regression analyses, the baseline outcome, recent physical activity, and resiliency were entered in the first step. Recent stressful life events, frequent group walks in nature, and the interaction term were entered in the second step with the enter method. All analyses were controlled for age and gender. Recent stressful life events and group walks in nature were centered at their means. Higher scores indicate greater perceived stress, depression, negative affect, positive affect, or mental well-being. ^a^ The Frequent Nature Group Walkers subsample consists of WfH participants from the main sample who attended a group walk in the natural environment at least once per week in the previous three months. ^b^ Propensity-matched sample; analysis weighted by the propensity score weight. ^c^ Physical activity in the previous week. ^d^ Recent stressful life events experienced in the previous three months: 0 = No stressful life events, 1 = One or more stressful life events. ^e^ Frequent group walk participation: 0 = Non-group Walkers, 1 = Frequent Nature Group Walkers. ^1^ Untransformed results are reported, as results for the untransformed data were similar to the log-transformed data. *B*, standardized coefficient; SE, standard error; β, standardized coefficient. * *p* < 0.05. ** *p* < 0.01. *** *p* < 0.001.

**Table 6:** Main effects model assessing frequent group walks in nature and recent stressful life events on perceived stress, depression, negative affect, positive affect, and mental well-being. Frequent Nature Group Walkers Subsample ^a^ (*n* = 930 ^b^).

	Perceived Stress			Depression ^1^			Negative Affect ^1^			Positive Affect			Mental Well-Being		
Final Step	*B*	*SE*	*β*	*B*	*SE*	*β*	*B*	*SE*	*β*	*B*	*SE*	*β*	*B*	*SE*	*β*
Baseline outcome	0.44	0.03	0.42 ***	0.51	0.03	0.53 ***	0.51	0.03	0.54 ***	0.53	0.03	0.50 ***	0.47	0.03	0.49 ***
Recent physical activity ^c^	−0.10	0.08	−0.03	−0.22	0.08	−0.06 **	−0.11	0.07	−0.04	0.39	0.09	0.10 ***	0.21	0.09	0.05 *
Resiliency	−0.34	0.03	−0.34 ***	−0.20	0.03	−0.20 ***	−0.17	0.02	−0.20 ***	0.31	0.03	0.26 ***	0.40	0.04	0.31 ***
Recent stressful life event ^d^	1.86	0.30	0.14 ***	1.27	0.31	0.10 ***	1.20	0.25	0.12 ***	−0.84	0.34	−0.05 *	−0.65	0.35	−0.04
Frequent group walks in nature ^e^	−1.26	0.32	−0.09 ***	−1.60	0.34	−0.12 ***	−0.72	0.27	−0.07 **	2.42	0.37	0.15 ***	1.54	0.38	0.09 ***
R^2^ for Step 1	0.52 ***			0.48 ***			0.49 ***			0.53 ***			0.57 ***		
Change R^2^	0.03 ***			0.02 ***			0.02 ***			0.03 ***			0.01 ***		

Note. The final step is shown. In all regression analyses, the baseline outcome, recent physical activity, and resiliency were entered in the first step. Recent stressful life events and frequent group walks in nature were entered in the second step, with the enter method. All analyses controlled for age and gender. Higher scores indicate greater perceived stress, depression, negative affect, positive affect, or mental well-being. ^a^ The Frequent Nature Group Walkers subsample consists of participants from the main sample who attended a group walk in the natural environment at least once per week in the previous three months. ^b^ Propensity-matched sample; analysis weighted by propensity score weight. ^c^ Physical activity in the previous week. ^d^ Recent stressful life events experienced in the previous three months: 0 = No stressful life events, 1 = One or more stressful life events. ^e^ Frequent Group walk participation: 0 = Non-group Walkers, 1 = Frequent Nature Group Walkers. ^1^ Untransformed results are reported, as results for the untransformed data were similar to the log-transformed data. *B*, standardized coefficient; SE, standard error; β, standardized coefficient. * *p* < 0.05. ** *p* < 0.01. *** *p* < 0.0001.

## Supplementary Materials

The following are available online at https://www.mdpi.com/1660-4601/16/6/986/s1. Table S1: Characteristics of the matcheda Nature Group Walkers (main sample) on demographics, health, and past stressful life events, Table S2: Characteristics of the matched Frequent Nature Group Walkers (subsample) on demographics, health status, past stressful life events and past physical activity, Table S3: Comparison of mean scores of social support, connectedness to nature and resiliency for matched Nature Group Walkers and Comparison Group (main sample) and matched Frequent Nature Group Walkers and Comparison group (subsample), Table S4: Number of recent stressful life events experienced by Nature Group Walkers or Propensity-matched Comparison group (main sample), Table S5: Number of recent stressful life events experienced by Frequent Nature Group Walkers or Propensity-matched Comparison group (subsample).

## References

[1] CDC Facts about Mental Health. https://www.cdc.gov/mentalhealth/learn/index.htm.

[2] World Health Organization Noncommunicable Diseases. http://www.euro.who.int/en/health-topics/noncommunicable-diseases.

[3] World Health Organization Mental Disorders. Fact Sheet. http://www.who.int/mediacentre/factsheets/fs396/en.

[4] National Institute of Mental Health Mental Illness. https://www.nimh.nih.gov/health/statistics/mental-illness.shtml.

[5] World Health Organization Prevalence of Mental Disorders. http://www.euro.who.int/en/health-topics/noncommunicable-diseases/mental-health/data-and-resources.

[6] Bloom D.E., Cafiero E.T., Jané-Llopis E. The Global Economic Burden of Noncommunicable Diseases. http://www3.weforum.org/docs/WEF_Harvard_HE_GlobalEconomicBurdenNonCommunicableDiseases_2011.pdf.

[7] Irvine K.N., Warber S.L. (2002). Greening Healthcare: Practicing as if the Natural Environment really Mattered. Altern. Ther. Health Med..

[8] Frumkin H., Bratman G.N., Breslow S.J., Cochran B., Kahn P.J., Lawler J.J., Levin P.S., Tandon P.S., Varanasi U., Wolf K.L. (2017). Nature Contact and Human Health: A Research Agenda. Environ. Health Perspect..

[9] Frumkin H. (2001). Beyond Toxicity: Human Health and the Natural Environment. Am. J. Prev. Med..

[10] Maller C., Townsend M., Pryor A., Brown P., St Leger L. (2005). Healthy Nature Healthy People: ‘Contact with Nature’ as an Upstream Health Promotion Intervention for Populations. Health Promot. Int..

[11] Ten Brink P., Mutafoglu K., Schweitzer J.P., Kettunen M., Twigger-Ross C., Baker J., Kuipers Y., Emonts M., Tyrväinen L., Hujala T. (2016). The Health and Social Benefits of Nature and Biodiversity Protection.

[12] World Health Organization (2016). Urban Green Spaces and Heatlh: A Review of Evidence.

[13] Van den Bosch M., Ode Sang Å. (2017). Urban Natural Environments as Nature-Based Solutions for Improved Public Health—A Systematic Review of Reviews. Environ. Res..

[14] Tyrväinen L., Ojala A., Korpela K., Lanki T., Tsunetsugu Y., Kagawa T. (2014). The Influence of Urban Green Environments on Stress Relief Measures: A Field Experiment. J. Environ. Psychol..

[15] Olafsdottir G., Cloke P., Schulz A., van Dyck Z., Eysteinsson T., Thorleifsdottir B., Vögele C. (2018). Health Benefits of Walking in Nature: A Randomized Controlled Study Under Conditions of Real-Life Stress. Environ. Behav..

[16] Berman M., Kross E., Krpan K., Askren M., Burson A., Deldin P., Kaplan S., Sherdell L., Gotlib I., Jonides J. (2012). Interacting with Nature Improves Cognition and Affect for Individuals with Depression. J. Affect. Disord..

[17] Department of Health (2011). Start Active, Stay Active: A Report on Physical Activity for Health from the Four Home Countries’ Chief Medical Officers.

[18] Robertson R., Robertson A., Jepson R., Maxwell M. (2012). Walking for Depression or Depressive Symptoms: A Systematic Review and Meta-Analysis. Ment. Health Phys. Act..

[19] Kelly P., Williamson C., Niven A.G., Hunter R., Mutrie N., Richards J. (2018). Walking on Sunshine: Scoping Review of the Evidence for Walking and Mental Health. Br. J. Sports Med..

[20] Hanson S., Jones A. (2015). Is there Evidence that Walking Groups have Health Benefits? A Systematic Review and Meta-Analysis. Br. J. Sports Med..

[21] Bowler D.E., Buyung-Ali L., Knight T., Pullin A.S. (2010). A Systematic Review of Evidence for the Added Benefits to Health of Exposure to Natural Environments. BMC Public Health.

[22] Thompson Coon J., Boddy K., Stein K., Whear R., Barton J., Depledge M.H. (2011). Does Participating in Physical Activity in Outdoor Natural Environments have a Greater Effect on Physical and Mental Wellbeing than Physical Activity Indoors? A Systematic Review. Environ. Sci. Technol..

[23] Roe J., Aspinall P. (2011). The Restorative Benefits of Walking in Urban and Rural Settings in Adults with Good and Poor Mental Health. Health Place.

[24] Marselle M.R., Irvine K.N., Warber S.L. (2013). Walking for Well-being: Are Group Walks in Certain Types of Natural Environments Better for Well-being than Group Walks in Urban Environments?. Int. J. Environ. Res. Public Health.

[25] Peacock J., Hine R., Pretty J. The Mental health benefits of green exercise activities and green care. https://psyk-info.regionsyddanmark.dk/dwn109161.pdf.

[26] Leppin A.L., Bora P.R., Tilburt J.C., Gionfriddo M.R., Zeballos-Palacios C., Dulohery M.M., Sood A., Erwin P.J., Brito J.P., Boehmer K.R. (2014). The Efficacy of Resiliency Training Programs: A Systematic Review and Meta-Analysis of Randomized Trials. PLoS ONE.

[27] American Psychological Association The Road to Resilience. http://www.apa.org/helpcenter/road-resilience.aspx.

[28] Mancini A.D., Bonanno G.A., Reich J.W., Zautra A.J., Hall J.S. (2010). Resilience to potential trauma: Toward a lifespan approach. Handbook of Adult Resilience.

[29] Bonanno G.A. (2004). Loss, Trauma, and Human Resilience: Have we underestimated the human capacity to thrive After extremely aversive events?. Am. Psychol..

[30] Ryff C., Singer B., Love G.D., Essex M.J., Lomranz J. (1998). Resilience in adulthood and later life: Defining features and dynamic processes. Handbook of Aging and Mental Health: An Integrative Approach.

[31] Masten A.S., Reed M.J., Synder C.R., Lopez S.J. (2005). Resilience in development. Handbook of Positive Psychology.

[32] Turner R.J., Wheaton B., Cohen S., Kessler R.C., Underwood Gordon L. (1997). Checklist measurement of stressful life events. Measuring Stress: A Guide for Health and Social Scientists.

[33] Kessler R. (1997). The effects of stressful life Events on depression. Annu. Rev. Psychol..

[34] Jordanova V., Stewart R., Goldberg D., Bebbington P.E., Brugha T., Singleton N., Lindesay J.E.B., Jenkins R., Prince M., Meltzer H. (2007). Age Variation in Life Events and their Relationship with Common Mental Disorders in a National Survey Population. Soc. Psychiatry Psychiatr. Epidemiol..

[35] Shevlin M., Houston J., Dorahy M., Adamson G. (2007). Cumulative Traumas and Psychosis: An Analysis of the National Comorbidity Survey and the British Psychiatric Morbidity Survey. Schizophr. Bull..

[36] Bebbington P.E., Hurry J., Tennant C. (1988). Adversity and the symptoms of depression. Int. J. Soc. Psychiatry.

[37] Zimmerman M.A., Ramirez-Valles J., Zapert K.M., Maton K.I. (2000). A Longitudinal Study of Stress-Buffering Effects for Urban African-American Male Adolescent Problem Behaviors and Mental Health. J. Community Psychol..

[38] Wardenaar K.J., van Veen T., Giltay E.J., Zitman F.G., Penninx B.W.J.H. (2014). The use of Symptom Dimensions to Investigate the Longitudinal Effects of Life Events on Depressive and Anxiety Symptomatology. J. Affect. Disord..

[39] Cohen S. (2000). Measures of Psychological Stress.

[40] Burns R.A., Machin M.A. (2013). Psychological Wellbeing and the Diathesis-Stress Hypothesis Model: The Role of Psychological Functioning and Quality of Relations in Subjective Well-being in a Life Events Study. Pers. Individ. Differ..

[41] Harrop E., Addis S., Elliott E., Williams G. Resilience, Coping and Salutogenic Approaches to Maintaining and Generating Health: A Review. https://www.nice.org.uk/guidance/ph6/evidence/behaviour-change-review-3-resilience-coping-and-salutogenic-approaches-to-maintaining-and-generating-health-pdf-369664527.

[42] Werner E.E. (1995). Resilience in Development. Curr. Dir. Psychol. Sci..

[43] Yates T.M., Masten A.S., Linley P.A., Joseph S. (2004). Fostering the future: Resilience theory and the practice of positive psychology. Positive Psychology in Practice.

[44] Luthar S.S., Cicchetti D., Becker B. (2000). The Construct of Resilience: A Critical Evaluation and Guidelines for Future Work. Child Dev..

[45] Zautra A.J., Hall J.S., Murray K.E., Reich J.W., Zautra A.J., Hall J.S. (2010). Resilience: A new definition of health for people and communities. Handbook of Adult Resilience.

[46] Masten A.S., Wright M.O., Reich J.W., Zautra A.J., Hall S. (2009). Resilience over the lifespan: Developmental perspectives on resistance, recovery, and transformation. Handbook of Adult Resilience.

[47] Kaplan R., Kaplan S. (1989). The Experience of Nature: A Psychological Perspective.

[48] Warber S.L., DeHudy A.A., Bialko M.F., Marselle M.R., Irvine K.N. (2015). Addressing “Nature-Deficit Disorder”: A Mixed Methods Pilot Study of Young Adults Attending a Wilderness Camp. Evid.-Based Complement. Altern. Med..

[49] Dallimer M., Davies Z.G., Irvine K.N., Maltby L.L., Warren P.H., Gaston K.J., Armsworth J.R. (2014). What Personal and Environmental Factors Determine Frequency of Urban Greenspace use?. Int. J. Environ. Res. Public Health.

[50] Fuller R.A., Irvine K.N., Devine-Wright P., Warren P.H., Gaston K.J. (2007). Psychological Benefits of Greenspace Increase with Biodiversity. Biol. Lett..

[51] Irvine K.N., Warber S.L., Devine-Wright P., Gaston K.J. (2013). Understanding Urban Green Space as a Health Resource: A Qualitative Comparison of Visit Motivation and Derived Effects among Park Users in Sheffield, UK. Int. J. Environ. Res. Public Health.

[52] Lemieux C.J., Eagles P.F., Slocombe D.S., Doherty S.T., Elliott S.J., Mock S.E. (2012). Human Health and Wellbeing Motivations and Benefits Associated with Protected Area Experiences: An Opportunity for Transforming Policy and Management in Canada. Parks Int. J. Prot. Areas Conserv..

[53] Block J., Kremen A.M. (1996). IQ and Ego-Resiliency: Conceptual and Empirical Connections and Separateness. J. Pers. Soc. Psychol..

[54] Ong A.D., Bergeman C.S., Bisconti T.L., Wallace K.A. (2006). Psychological Resilience, Positive Emotions, and Successful Adaptation to Stress in Later Life. J. Pers. Soc. Psychol..

[55] Almeida D.M. (2005). Resilience and Vulnerability to Daily Stressors Assessed Via Diary Methods. Curr. Dir. Psychol. Sci..

[56] Synder C.R., Lopez S.J. (2007). Positive Psychology: The Scientific and Practical Explorations of Human Strengths.

[57] Friedli L. (2009). Mental Health, Resilience and Inequalities. http://www.euro.who.int/__data/assets/pdf_file/0012/100821/E92227.pdf.

[58] Slopen N., Non A., Williams D.R., Roberts A.L., Albert M.A. (2014). Childhood Adversity, Adult Neighborhood Context, and Cumulative Biological Risk for Chronic Diseases in Adulthood. Psychosom. Med..

[59] Hartig T., Fernández-Ramírez B., Hidalgo Villodres C., Salvador Ferrer C.M., Martos Méndez M.J. (2011). Issues in restorative environments research: Matters of measurement. Psicología Ambiental 2011: Entre Los Estudios Urbanos y El Análisis De La Sostenibilidad [Environmental Psychology 2011: Between Urban Studies and the Analysis of Sustainability].

[60] Ulrich R., Simons R., Losito B., Fiorito E., Miles M., Zelson M. (1991). Stress Recovery during Exposure to Natural and Urban Environments. J. Environ. Psychol..

[61] Ulrich R., Altman I., Wohlwill J. (1983). Aesthetic and affective response to natural environment. Human Behavior and the Natural Environment.

[62] Van den Berg M.M.H.E., Maas J., Muller R., Braun A., Kaandorp W., van Lien R., an Poppel M.N.M., van Mechelen W., van den Berg A.E. (2015). Autonomic Nervous System Responses to Viewing Green and Built Settings: Differentiating between Sympathetic and Parasympathetic Activity. Int. J. Environ. Res. Public Health.

[63] Parsons R., Tassinary L., Ulrich R., Hebl M., Grossman-Alexander M. (1998). The View from the Road: Implications for Stress Recovery and Immunization. J. Environ. Psychol..

[64] Lottrup L., Grahn P., Stigsdotter U.K. (2013). Workplace Greenery and Perceived Level of Stress: Benefits of Access to a Green Outdoor Environment at the Workplace. Landsc. Urban Plan..

[65] Triguero-Mas M., Gidlow C., Martínez D., de Bont J., Carrasco-Turigas G., Martinez-Iniguez T., Hurst G., Masterson D., Donaire-Gonzalez D., Seto E. (2017). The Effect of Randomised Exposure to Different Types of Natural Outdoor Environments Compared to Exposure to an Urban Environment on People with Indications of Psychological Distress in Catalonia. PLoS ONE.

[66] Kaplan S. (1995). The Restorative Benefits of Nature: Toward an Integrative Framework. J. Environ. Psychol..

[67] Kaplan S., Berman M.G. (2017). Directed Attention as a Common Resource for Executive Functioning and Self-Regulation. Perspect. Psychol. Sci..

[68] Kuo F.E. (2001). Coping with Poverty—Impacts of Environment and Attention in the Inner City. Environ. Behav..

[69] Berman M., Jonides J., Kaplan S. (2008). The Cognitive Benefits of Interacting with Nature. Psychol. Sci..

[70] Herzog T.R. (1997). Reflection and Attentional Recovery as Distinctive Benefits of Restorative Environments. J. Environ. Psychol..

[71] Crane M., Boga D. (2017). Rethinking Approaches to Resilience and Mental Health Training. J. Mil. Veterans’ Health.

[72] Grant L., Kinman G. (2013). The Importance of Emotional Resilience for Staff and Students in the ‘Helping’ Professions: Developing an Emotional Curriculum.

[73] Joseph S. (2012). What Doesn’t Kill US. Psychologist.

[74] Fredrickson L.M., Anderson D.H. (1999). A Qualitative Exploration of the Wilderness Experience as a Source of Spiritual Inspiration. J. Environ. Psychol..

[75] Sonntag-Öström E., Stenlund T., Nordin M., Lundell Y., Ahlgren C., Fjellman-Wiklund A., Järvholm L.S., Dolling A. (2015). “Nature’s Effect on My Mind”—Patients’ Qualitative Experiences of a Forest-Based Rehabilitation Programme. Urban For. Urban Green..

[76] Naor L., Mayseless O. (2017). How Personal Transformation Occurs Following a Single Peak Experience in Nature: A Phenomenological Account. J. Hum. Psychol..

[77] Masten A.S. (2001). Ordinary Magic. Am. Psychol..

[78] Masten A.S., Obradovic J. (2006). Competence and Resilience in Development. Ann. N. Y. Acad. Sci..

[79] Baron R.M., Kenny D.A. (1986). The Moderator-Mediator Variable Distinction in Social Psychological Research: Conceptual, Strategic, and Statistical Considerations. J. Pers. Soc. Psychol..

[80] Hayes A.F. (2018). Partial, Conditional, and Moderated Moderated Mediation: Quantification, Inference, and Interpretation. Commun. Monogr..

[81] Wells N.M., Evans G.W. (2003). Nearby Nature: A Buffer of Life Stress among Rural Children. Environ. Behav..

[82] Corraliza J.A., Collado S. (2011). La Naturaleza Cercana Como Moderadora Del Estrés Infantil. Psicothema.

[83] Leather P., Pyrgas M., Beale D., Lawrence C. (1998). Windows in the Workplace: Sunlight, View, and Occupational Stress. Environ. Behav..

[84] Van den Berg A.E., Maas J., Verheij R.A., Groenewegen P.P. (2010). Green Space as a Buffer between Stressful Life Events and Health. Soc. Sci. Med..

[85] Stuart S.M., Barlett P.F. (2005). Lifting spirits: Creating gardens in California domestic violence shelters. Urban Place: Reconnecting with the Natural World.

[86] Sahlin E., Ahlborg G., Matuszczyk J.V., Grahn P. (2014). Nature-Based Stress Management Course for Individuals at Risk of Adverse Health Effects from Work-Related Stress—Effects on Stress Related Symptoms, Workability and Sick Leave. Int. J. Environ. Res. Public Health.

[87] Ottosson J., Grahn P. (2008). The Role of Natural Settings in Crisis Rehabilitation: How does the Level of Crisis Influence the Response to Experiences of Nature with Regard to Measures of Rehabilitation?. Landsc. Res..

[88] Cimprich B., Ronis D. (2003). An Environmental Intervention to Restore Attention in Women with Newly Diagnosed Breast Cancer. Cancer Nurs..

[89] South J., Giuntoli G., Kinsella K (2013). An Evaluation of the Walking for Wellness Project and the Befriender Role (Report Number 118). Natural England: Peterborough. http://publications.naturalengland.org.uk/publication/4853061788893184.

[90] Marselle M.R., Irvine K.N., Warber S.L. (2014). The Effects of Group Walks in Nature on Multiple Dimensions of Well-being. Ecopsychology.

[91] Shanahan D.F., Bush R., Gaston K.J., Lin B.B., Dean J., Barber E., Fuller R.A. (2016). Health Benefits of Nature Experiences Depend on Dose. Sci. Rep..

[92] France J., Sennett J., Jones A. (2016). Evaluation of Walking for Health. Final Report to Macmillan and the Ramblers. March 2016.

[93] Walking for Health About Us, n.d. https://www.walkingforhealth.org.uk/about-us.

[94] Fitches T. Who Took Part in Walking for Health? (Report Number NERR041). http://publications.naturalengland.org.uk/publication/35027.

[95] Cohen S., Kamarck T., Mermelstein R. (1983). A Global Measure of Perceived Stress. J. Health Soc. Behav..

[96] Olsen L., Mortensen E., Bech P. (2004). Prevalence of Major Depression and Stress Indicators in the Danish General Population. Acta Psychiatr. Scand..

[97] Watson D., Clark L.A., Tellegen A. (1988). Development and Validation of Brief Measures of Positive and Negative Affect: The PANAS Scales. J. Pers. Soc. Psychol..

[98] Tennant R., Hiller L., Fishwick R., Platt S., Joseph S., Weich S., Parkinson J., Secker J., Stewart-Brown S. (2007). The Warwick-Edinburgh Mental Well-being Scale (WEMWBS): Development and UK Validation. Health Qual. Life Outcomes.

[99] Tusaie K., Dyer J. (2004). Resilience: A Historical Review of the Construct. Holist. Nurs. Pract..

[100] Seery M.D., Holman E.A., Silver R.C. (2010). Whatever does Not Kill Us: Cumulative Lifetime Adversity, Vulnerability, and Resilience. J. Pers. Soc. Psychol..

[101] Brugha T., Cragg D. (1990). The List of Threatening Experiences: The Reliability and Validity of a Brief Life Events Questionnaire. Acta Psychiatr. Scand..

[102] Brugha T., Bebbington P., Tennant C., Hurry J. (1985). The List of Threatening Experiences: A Subset of 12 Life Event Categories with Considerable Long-Term Contextual Threat. Psychol. Med..

[103] Singleton N., Lee A., Meltzer H., Office for National Statistics (2002). Appendix B: The Questionnaire. Psychiatric Morbidity among Adults Living in Private Households, 2000: Technical Report.

[104] Childs E., de Wit H. (2014). Regular Exercise is Associated with Emotional Resilience to Acute Stress in Healthly Adults. Front. Psychol..

[105] Milton K., Bull F., Bauman A. (2011). Reliability and Validity Testing of a Single-Item Physical Activity Measure. Br. J. Sports Med..

[106] Cohen S. (2004). Social Relationships and Health. Am. Psychol..

[107] Zimmerman M.A., Brenner A.B., Reich J.W., Zautra A.J., Hall J.S. (2010). Resilience in adolescence: Overcoming neighborhood disadvantage. Handbook of Adult Resilience.

[108] Cohen S., Mermelstein R., Kamarck T., Hoberman H., Sarason I.G., Sarason B.R. (1985). Measuring the functional components of social support. Social Support: Theory, Research and Application.

[109] Capaldi C.A., Dopko R.L., Zelenski J.M. (2014). The Relationship between Nature Connectedness and Happiness: A Meta-Analysis. Front. Psychol..

[110] Mayer F.S., Frantz C.M., Bruehlman-Senecal E., Dolliver K. (2009). Why is Nature Beneficial? the Role of Connectedness to Nature. Environ. Behav..

[111] Wensley R., Slade A. (2012). Walking as a Meaningful Leisure Occupation: The Implications for Occupational Therapy. Br. J. Occup. Ther..

[112] Mayer F.S., Frantz C.M. (2004). The Connectedness to Nature Scale: A Measure of Individuals’ Feeling in Community with Nature. J. Environ. Psychol..

[113] Burns R., Anstey K. (2010). The Connor-Davidson Resilience Scale (CD-RISC): Testing the Invariance of a Uni-Dimensional Resilience Measure that is Independent of Positive and Negative Affect. Pers. Individ. Differ..

[114] Fredrickson B., Tugade M., Waugh C., Larkin G. (2003). What Good are Positive Emotions in Crises? A Prospective Study of Resilience and Emotions Following the Terrorist Attacks on the United States on September 11th, 2001. J. Pers. Soc. Psychol..

[115] Tugade M.M., Fredrickson B.L. (2004). Resilient Individuals use Positive Emotions to Bounce Back from Negative Emotional Experiences. J. Pers. Soc. Psychol..

[116] Smith B.W., Tooley E.M., Christopher P.J., Kay V.S. (2010). Resilience as the Ability to Bounce Back from Stress: A Neglected Personal Resource?. J. Posit. Psychol..

[117] Smith B.W., Dalen J., Wiggins K., Tooley E., Christopher P., Bernard J. (2008). The Brief Resilience Scale: Assessing the Ability to Bounce Back. Int. J. Behav. Med..

[118] Wingo A.P., Wrenn G., Pelletier T., Gutman A.R., Bradley B., Ressler K.J. (2010). Moderating Effects of Resilience on Depression in Individuals with a History of Childhood Abuse or Trauma Exposure. J. Affect. Disord..

[119] Buchecker M., Degenhardt B. (2015). The Effects of Urban Inhabitants’ Nearby Outdoor Recreation on their Wellbeing and their Psychological Resilience. J. Outdoor Recreat. Tour..

[120] Campbell-Sills L., Stein M. (2007). Psychometric Analysis and Refinement of the Connor-Davidson Resilience Scale (CD-RISC): Validation of a 10 Item Measure of Resilience. J. Trauma. Stress.

[121] Connor K.M., Davidson J.R.T. (2003). Development of a New Resilience Scale: The Connor-Davidson Resilience Scale (CD-RISC). Depression and Anxiety.

[122] Department for Communities and Local Government The English Indices of Deprivation. https://www.gov.uk/government/statistics/english-indices-of-deprivation-2010.

[123] Walking for Health (2013). Outdoor Health Questionnaire. http://www.wandsworth.gov.uk/download/downloads/id/9421/outdoor_health_questionnaire.pdf.

[124] Rosenbaum P.R., Rubin D.B. (1983). The Central Role of the Propensity Score in Observational Studies for Causal Effects. Biometrika.

[125] Craig P., Cooper C., Gunnell D. (2011). Using Natural Experiments to Evaluate Population Health Interventions: Guidance for Producers and Users of Evidence.

[126] Boer R., Zheng Y., Overton A., Ridgeway G.K., Cohen D.A. (2007). Neighborhood Design and Walking Trips in Ten U.S. Metropolitan Areas. Am. J. Prev. Med..

[127] Cohen D.A., Lapham S., Evenson K.R., Williamson S., Golinelli D., Ward P., Hillier A., McKenzie T.L. (2013). Use of Neighbourhood Parks: Does Socio-Economic Status Matter? A Four-City Study. Public Health.

[128] Hendriksen I.J.M., Simons M., Garre F.G., Hildebrandt V.H. (2010). The Association between Commuter Cycling and Sickness Absence. Prev. Med..

[129] Ho D.E., Imai K., King G., Stuart E.A. (2007). Matching as Nonparametric Preprocessing for Reducing Model Dependence in Parametric Causal Inference. Political Anal..

[130] Stuart E.A., Rubin D.B. Matching Methods for Causal Inference: Designing Observational Studies. http://www.biostat.jhsph.edu/~estuart/StuRub_MatchingChapter_07.pdf.

[131] Stuart E.A. (2010). Matching Methods for Causal Inference: A Review and a Look Forward. Stat. Sci..

[132] Dehejia R.H., Wahba S. (1999). Causal Effects in Nonexperimental Studies: Reevaluating the Evaluation of Training Programs. J. Am. Stat. Assoc..

[133] Thoemmes (2012). Propensity Score Matching in SPSS. http://arxiv.org/pdf/1201.6385.pdf.

[134] Maletta H. (2007). Weighting. http://www.spsstools.net/Tutorials/WEIGHTING.pdf.

[135] UCLA: Statistical Consulting Group (2013). What Types of Weights do SAS, Stata and SPSS Support?. http://www.ats.ucla.edu/stat/stata/faq/weights.htm.

[136] IBM (2012). Compare Means and Univariate Give Different Results with Fractional Weights. http://www-01.ibm.com/support/docview.wss?uid=swg21592441.

[137] Tabachnick B.G., Fidell L.S. (2013). Using Multivariate Statistics.

[138] Evans G.W., Lepore S.J., Moore G.T., Marans R.W. (1997). Moderating and mediating processes in environment-behavior research. Advances in Environment, Behavior and Design. Volume 4: Toward an Integration of Theory, Methods, Research, and Utilization.

[139] Hoyle R.H., Robinson J.C., Sansone C., Morf C.C., Panter A.T. (2004). Mediated and moderated effects in social psychological research: Measurement, design, and analysis issues. The Sage Handbook of Methods in Social Psychology.

[140] Miles J., Shevlin M. (2001). Applying Regression and Correlation: A Guide for Students and Researchers.

[141] Field A. (2013). Discovering Statistics using IBM SPSS Statistics.

[142] Hayes A.F. (2013). Introduction to Mediation, Moderation, and Conditional Process Analysis: A Regression-Based Approach.

[143] Cohen S., Wills T.A. (1985). Stress, Social Support, and the Buffering Hypothesis. Psychol. Bull..

[144] Holmes G., Evans N. Walk and Talk. Proceedings of the 1st International Conference on the Multi-Dimensional Aspects of Wellbeing.

[145] Staats H., van Gemerden E., Hartig T. (2010). Preference for Restorative Situations: Interactive Effects of Attentional State, Activity-in-Environment, and Social Context. Leis. Sci..

[146] Korpela K., Staats H., Coplan R.J., Bowker J.C. (2014). Restorative qualities of being alone in nature. The Handbook of Solitude: Psychological Perspectives on Social Isolation, Social Withdrawal, and being Alone.

[147] Wohlwill J.F. (1983). The Concept of Nature: A Psychologist’s View. Hum. Behav. Environ. Adv. Theory Res..

[148] Staats H., Hartig T. (2004). Alone or with a Friend: A Social Context for Psychological Restoration and Environmental Preferences. J. Environ. Psychol..

[149] Hynds H. (2009). and Allibone, C. What Motivates People to Participate in Organised Walking Activity? (Report Number NERR028).

[150] White M.P., Pahl S., Ashbullby K., Herbert S., Depledge M.H. (2013). Feelings of Restoration from Recent Nature Visits. J. Environ. Psychol..

[151] Krzywicka P., Byrka K. (2017). Restorative Qualities of and Preference for Natural and Urban Soundscapes. Front. Psychol..

[152] Johansson M., Hartig T., Staats H. (2011). Psychological Benefits of Walking: Moderation by Company and Outdoor Environment. Appl. Psychol. Health Well-Being.

[153] Priest P. (2007). The Healing Balm Effect: Using a Walking Group to Feel Better. J. Health Psychol..

[154] Barton J., Griffin M., Pretty J. (2012). Exercise-, Nature- and Socially Interactive-Based Initiatives to Improve Mood and Self-Esteem in the Clinical Population. Perspect. Public Health.

[155] Iwata Y., Ni Dhubhadin A., Brophy J., Roddy D., Burke C., Murphy B. (2016). Benefits of Group Walking in Forests for People with Significant Mental Ill-Health. Ecopsychology.

[156] Marselle M.R., Irvine K.N., Lorenzo-Arribas A., Warber S.L. (2016). Does Perceived Restorativeness Mediate the Effects of Perceived Biodiversity and Perceived Naturalness on Emotional Well-being Following Group Walks in Nature?. J. Environ. Psychol..

[157] Harder V.S., Stuart E.A., Anthony J.C. (2010). Propensity Score Techniques and the Assessment of Measured Covariate Balance to Test Causal Associations in Psychological Research. Psychol. Methods.

[158] Bielinis E., Takayama N., Boiko S., Omelan A., Bielinis L. (2018). The Effect of Winter Forest Bathing on Psychological Relaxation of Young Polish Adults. Urban For. Urban Green..

[159] Keniger L.E., Gaston K.J., Irvine K.N., Fuller R.A. (2013). What are the Benefits of Interacting with Nature?. Int. J. Environ. Res. Public Health.

[160] Bratman G.N., Daily G.C., Levy B.J., Gross J.J. (2015). The Benefits of Nature Experience: Improved Affect and Cognition. Landsc. Urban Plan..

[161] Bratman G.N., Hamilton J.P., Hahn K.S., Daily G.C., Gross J.J. (2015). Nature Experience Reduces Rumination and Subgenual Prefrontal Cortex Activation. PNAS.

[162] Gonzalez M.T., Hartig T., Patil G.G., Martinsen E.W., Kirkevold M. (2010). Therapeutic Horticulture in Clinical Depression: A Prospective Study of Active Components. J. Adv. Nurs..

[163] Wendel-Vos W., Droomers M., Kremers S., Brug J., van Lenthe F. (2007). Potential Environmental Determinants of Physical Activity in Adults: A Systematic Review. Obes. Rev..

[164] Ball K., Bauman A., Leslie E., Owen N. (2001). Perceived Environmental Aesthetics and Convenience and Company are Associated with Walking for Exercise among Australian Adults. Prev. Med..

[165] Lovell R., Husk K., Blockley K., Bethel A., Bloomfield D., Warber S., Pearson M., Lang I., Byng R., Garside R. (2017). A Realist Review and Collaborative Development of what Works in the Social Prescribing Process. Lancet.

[166] Bickerdike L., Booth A., Wilson P.M., Farley K., Wright K. (2017). Social Prescribing: Less Rhetoric and More Reality. A Systematic Review of the Evidence. BMJ Open.

[167] Cook P.A., Howarth M., Wheater C.P., Marselle M.R., Stadler J., Korn H., Irvine K.N., Bonn A. (2019). Biodiversity and health in the face of climate change—Implications for public health. Biodiversity and Health in the Face of Climate Change.

