# Supplementary material for: Growing Resilience through Interaction with Nature: Can Group Walks in Nature Buffer the Effects of Stressful Life Events on Mental Health?
Source: Int J Environ Res Public Health. 2019 Mar 19;16(6):986. doi: 10.3390/ijerph16060986 (PMC6466337; doi:10.3390/ijerph16060986)
Supplement: Supplementary file 1 [file ijerph-16-00986-s001.pdf]

Supplementary Files

Growing Resilience through Interaction with Nature: Can Group Walks in Nature Buffer the Effects of Stressful Life Events on Mental Health?

Melissa R. Marselle [1,2,*], Sara L. Warber [3,4] and Katherine N. Irvine [5]

[1] Department of Ecosystem Services, Helmholtz Centre for Environmental Research –UFZ, , Permoserstr. 15, 04318 Leipzig, Germany
[2] German Centre for Integrative Biodiversity Research (iDiv) Halle-Jena-Leipzig, Deutscher Platz 5e, 04103 Leipzig, Germany
[3] Department of Family Medicine, University of Michigan, Ann Arbor, MI, 48104, USA; swarber@med.umich.edu
[4] European Centre for Environment and Human Health, University of Exeter School of Medicine, Truro, TR1 3HD, UK
[5] Social, Economic and Geographical Sciences, The James Hutton Institute, Craigiebuckler, Aberdeen, Scotland, AB15 8QH, UK; Katherine.Irvine@hutton.ac.uk
* Correspondence: melissa.marselle@ufz.de; Tel.: +49-341-9733151

Table S1. Characteristics of the matched[a] Nature Group Walkers (main sample) on demographics, health, and past stressful life events.

**Demographics, health status, and past stressful life events and physical activity**	**Nature group walkers (n = 1081)**	**Comparison Group (n = 435)**	**Statistic**	***p*-value**
Gender (female %)	65.5%	68.1%	X^2 (1) = 0.952	0.329
Age (55+ %)	88.3%	88.4%	X^2 (1) = 0.000	0.995
Marital status (in a relationship %)	71.3%	70.4%	X^2 (1) = 0.122	0.727
Qualification (tertiary education %)	52.2%	54.9%	X^2 (2) = 3.560	0.169
Ethnicity (white %)	96.7%	97.2%	X^2 (1) = .314	0.575
Deprivation (least deprived %)	50.9%	51.5%	X^2 (2) = .224	0.894
Health condition (with condition) %	16.2%	20.5%	X^2 (1) = 4.039	0.044
Medical condition (with condition) %	34.8%	38.5%	X^2 (1) = 1.904	0.168
Disability (with disability) %	8.9%	6.7%	X^2 (1) = 2.033	0.154
GP recommend WfH (yes) %	6.2%	7.9%	X^2 (1) = 1.369	0.242
Past stressful life events[b] (none) %	32.9%	32.5%	X^2 (2) = 0.061	0.970
Past Physical activity[c] *M* (SD)	3.50 (1.94)	3.32 (2.13)	t(740.02) = 1.502	0.134

Note. Data reproduced from Marselle et al. (2014). [a] Propensity matched sample; all analyses weighted by propensity score weight. [b] Stressful life events experienced in the past year. [c] Number of days in the week prior to first WfH group walk took part in 30 minutes or more of physical activity.

Table S2. Characteristics of the matched [a] Frequent Nature Group Walkers [b] (subsample) on demographics, health status, past stressful life events and past physical activity.

**Demographics, health status, and past stressful life events and physical activity**	**Frequent green group walkers (*n* = 631)**	**Comparison Group (*n* = 306)**	**Statistic**	***p*-value**
Gender (female %)	62.8%	61%	X^2 (1) = 0.266	0.606
Age (55+ %)	92.7%	92.7%	X^2 (1) = 0.000	1.00
Marital status (in a relationship %)	71.6%	71.6%	X^2 (1) = 0.000	1.00
Qualification (tertiary education %)	46%	46%	X^2 (2) = 0.968	0.616
Ethnicity (white %)	96.5%	96.2%	X^2 (1) = 0.058	0.810
Deprivation (least deprived %)	49.4%	49.1%	X^2 (2) = 0.178	0.915
Health condition (with condition) %	17.6%	18.4%	X^2 (1) = 0.088	0.766
Medical condition (with condition) %	36.6%	38.5%	X^2 (1) = 0.319	0.572
Disability (with disability) %	9%	9%	X^2 (1) = 0.000	1.00
GP recommend WfH (yes) %	7%	9.2%	X^2 (1) = 1.428	0.232
Past stressful life events[c] (none) %	33.3%	34.7%	X^2 (2) = 2.552	0.279
Past Physical activity[d] *M* (SD)	3.54 (1.89)	3.49 (1.96)	t(935) = -0.357	0.721

Note. [a] Propensity matched sample; all analyses weighted by propensity score weight. [b] The Frequent Nature Group Walkers subsample consists of participants from the main sample who attended a group walk in the natural environment at least once per week in the previous three months. [c] Stressful life events experienced in the past year. [d] Number of days in the week prior to first WfH group walk took part in 30 minutes or more of physical activity.

Table S3. Comparison of mean scores of social support, connectedness to nature and resiliency for matched [a] Nature Group Walkers and Comparison Group (main sample) and matched [a] Frequent Nature Group Walkers [b] and Comparison group (subsample).

**Outcomes[c]**	**Nature Group Walkers (*M* (SD))**	**Comparison Group (*M* (SD))**	***t*-test[d]**	***p*-value**
**Nature Group Walkers (Main sample)[1]**				
Recent physical activity[e]	3.46 (1.79)	2.94 (2.11)	$t(689.96) = -4.50$	< 0.001
Social support	22.94 (6.44)	22.82 (6.47)	$t(1514) = -0.33$	0.74
Connectedness to nature[e]	51.75 (7.67)	50.79 (8.51)	$t(732.47) = -2.05$	0.04
Resiliency[e]	28.60 (6.05)	27.85 (6.96)	$t(711.87) = -1.97$	0.05
**Frequent Nature Group Walkers (Subsample)[2]**				
Recent physical activity[e]	3.65 (1.76)	3.03 (2.06)	$t(523.88) = -4.52$	< 0.001
Social support	22.83 (6.57)	22.36 (6.50)	$t(935) = -1.02$	0.31
Connectedness to nature[e]	51.51 (7.62)	51.24 (8.29)	$t(560.75) = -0.49$	0.63
Resiliency[e]	28.68 (6.06)	27.73 (6.93)	$t(537.24) = -2.05$	0.04

Note. [a]Propensity score matched sample; analysis weighted by propensity score weight. [b] The Frequent Nature Group Walkers subsample consists of participants from the main sample who attended a group walk in the natural environment at least once per week in the previous three months.[c] Higher scores indicate greater: social support (range 0-30), connection to nature (range 14-70), and resiliency (range 0-40). [d] Independent samples *t*-test. [e] Equal variances not assumed. [1]Nature Group Walkers $n = 1081$; Comparison Group $n = 435$. [2] Frequent Nature Group Walkers $n = 631$; Comparison Group $n = 306$.

Table S4. Number of recent stressful life events experienced by Nature Group Walkers or Propensity-matched Comparison group (main sample).

**Stressful life events**	**Comparison group (*n* = 435)**	**Nature Group Walkers (*n* = 1081)**
No life event	56.9% (*n* = 248)	58.1% (*n* = 628)
1 or more life event	43.1% (*n* = 187)	41.9% (*n* = 453)
M (SD)	0.43 (0.50)	0.42 (0.49)

Note. Propensity score matched sample; analysis weighted by propensity score weight. There was a no significant difference between number of stressful life events experienced and group membership, $t(1514) = 0.414$, $p = 0.68$.

Table S5. Number of recent stressful life events experienced by Frequent Nature Group Walkers or Propensity-matched Comparison group (subsample).

**Stressful life events**	**Comparison group (*n* = 306)**	**Frequent Nature Group Walkers (*n* = 631)**
No life event	51.8% (*n* = 159)	58.8% (*n* = 371)
1 or more life event	48.2% (*n* = 147)	41.2% (*n* = 260)
M (SD)	0.48 (0.50)	0.41 (0.49)

Note. Propensity score matched sample; analysis weighted by propensity score weight. There was a marginally significant difference between number of stressful life events experienced and group membership, $t(595.27) = 2.010$, $p = 0.05$.
